# Blockchain-Based Security Mechanisms for IoMT Edge Networks in IoMT-Based Healthcare Monitoring Systems

**DOI:** 10.3390/s22072449

**Published:** 2022-03-22

**Authors:** Filippos Pelekoudas-Oikonomou, Georgios Zachos, Maria Papaioannou, Marcus de Ree, José C. Ribeiro, Georgios Mantas, Jonathan Rodriguez

**Affiliations:** 1 Instituto de Telecommunicaçoes, 3810-193 Aveiro, Portugal; g.zachos@av.it.pt (G.Z.); m.papaioannou@av.it.pt (M.P.); mderee@av.it.pt (M.d.R.); jcarlosvgr@av.it.pt (J.C.R.); gimantas@av.it.pt (G.M.); jonathan@av.it.pt (J.R.); 2 Faculty of Engineering and Science, University of Greenwich, Chatham Maritime ME4 4TB, UK; 3 Faculty of Computing, Engineering and Science, University of South Wales, Pontypridd CF37 1DL, UK

**Keywords:** IoMT, blockchain, authentication, authorization, anomaly-based IDS, healthcare

## Abstract

Despite the significant benefits that the rise of Internet of Medical Things (IoMT) can bring into citizens’ quality of life by enabling IoMT-based healthcare monitoring systems, there is an urgent need for novel security mechanisms to address the pressing security challenges of IoMT edge networks in an effective and efficient manner before they gain the trust of all involved stakeholders and reach their full potential in the market of next generation IoMT-based healthcare monitoring systems. In this context, blockchain technology has been foreseen by the industry and research community as a disruptive technology that can be integrated into novel security solutions for IoMT edge networks, as it can play a significant role in securing IoMT devices and resisting unauthorized access during data transmission (i.e., tamper-proof transmission of medical data). However, despite the fact that several blockchain-based security mechanisms have already been proposed in the literature for different types of IoT edge networks, there is a lack of blockchain-based security mechanisms for IoMT edge networks, and thus more effort is required to be put on the design and development of security mechanisms relying on blockchain technology for such networks. Towards this direction, the first step is the comprehensive understanding of the following two types of blockchain-based security mechanisms: (a) the very few existing ones specifically designed for IoMT edge networks, and (b) those designed for other types of IoT networks but could be possibly adopted in IoMT edge networks due to similar capabilities and technical characteristics. Therefore, in this paper, we review the state-of-the-art of the above two types of blockchain-based security mechanisms in order to provide a foundation for organizing research efforts towards the design and development of reliable blockchain-based countermeasures, addressing the pressing security challenges of IoMT edge networks in an effective and efficient manner.

## 1. Introduction

The Internet of Things (IoT) technology has emerged and grown rapidly in the last few years, bringing significant benefits to the healthcare sector by transforming the healthcare industry itself and introducing the Internet of Medical Things (IoMT), where medical devices are interconnected in a way that anyone, anywhere, and anytime may have access to [1,2]. The evolution and rise of IoMT can play a noteworthy role in improving citizens’ quality of life by enabling IoMT-based healthcare monitoring systems that provide personalized and user-centric healthcare services overcoming constraints such as time and location [3]. Nevertheless, the wide range of different communication technologies (e.g., WLANs, Bluetooth, Zigbee) and types of IoMT devices (e.g., bio sensors, actuators, wireless access points) in IoMT-based healthcare monitoring systems, as well as the fact that the transmission between patients and healthcare providers of personal and confidential healthcare information (e.g., patient’s personal details and vital signs) is done through the internet, are factors that raise many security and privacy challenges [4,5,6,7]. Thus, security solutions that meet the fundamental security requirements (i.e., authentication, authorization/access control, data integrity, data confidentiality, and availability) for IoMT-based healthcare monitoring systems are essential for the acceptance and wide adoption of such systems in the coming next years. Nevertheless, the high resource requirements of complex and heavyweight conventional security mechanisms cannot be afforded by resource-constrained IoMT edge networks which constitute the key underlying components of IoMT-based healthcare monitoring systems [2]. In addition, the centralization approach widely adopted by the state-of-the-art security frameworks is not well applicable to IoMT edge networks due to single point of failure issues [8,9,10]. Last but not least, it is worthwhile highlighting that conventional state-of-the-art defence mechanisms cannot ensure complete tamper-proof systems for protecting IoMT edge networks [5]. Therefore, there is an urgent need for novel security mechanisms to address the pressing security challenges of IoMT edge networks in an effective and efficient manner before they gain the trust of all involved stakeholders and reach their full potential in the healthcare market [4,5]. In this context, blockchain technology has been foreseen by the industry and research community as a disruptive technology that can be integrated into novel security solutions for IoMT edge networks, as it can play a significant role in: (a) securing IoMT devices and (b) resisting unauthorized access during data transmission (i.e., tamper-proof transmission of medical data) [11]. However, despite the fact that several blockchain-based security mechanisms have already been proposed in the literature for different types of IoT edge networks, there is a lack of blockchain-based security mechanisms for IoMT edge networks, and thus more effort is required to be put on the design and development of security mechanisms relying on blockchain technology for such networks. Towards this direction, the first step is the comprehensive understanding of the following two types of blockchain-based security mechanisms: (a) the very few existing ones specifically designed for IoMT edge networks, and (b) those designed for other types of IoT networks but could be possibly adopted in IoMT edge networks due to similar capabilities and technical characteristics. Therefore, in this paper, we review the state-of-the-art of the above two types of blockchain-based security mechanisms in order to provide a foundation for organizing research efforts towards the design and development of reliable blockchain-based countermeasures addressing the pressing security challenges of IoMT edge networks in an effective and efficient manner. It is worth mentioning that we narrowed our focus down on the integration of blockchain technology into: (a) authentication and authorization mechanisms, as both comprise the first level of effective security in any system, and (b) Anomaly-based Intrusion Detection Systems (AIDSs), leveraging Machine Learning (ML) techniques, because of their ability to detect new, previously unknown attacks [11,12,13,14].

Following the introduction, this paper is organized as follows. In Section 2, the architecture and key components of an IoMT-based healthcare system are presented. Section 3 discusses the most popular consensus mechanisms for blockchain-based IoT applications and gives an overview of the main blockchain implementation platforms used in industry and academia. In Section 4, we examine the few existing blockchain-based security mechanisms specifically designed for IoMT edge networks, and blockchain-based security mechanisms which have been designed for other types of IoT networks but could be possibly adopted in IoMT edge networks. Finally, Section 5 concludes the paper and provides an insight for our future plans.

## 2. IoMT-Based Healthcare Monitoring Systems

**Figure 1:**
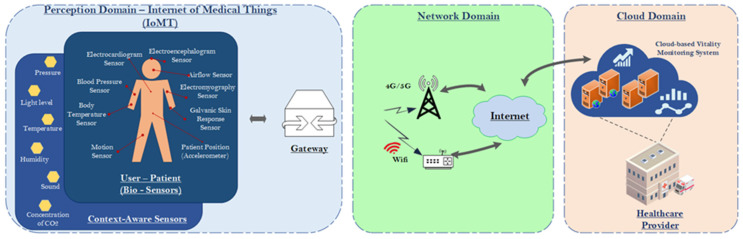
IoMT edge network architecture and key components.

Typically, an IoMT-based health monitoring system consists of the following domains: (a) perception domain; (b) network domain; and (c) cloud domain, as shown in Figure 1. The perception domain can be perceived as the device layer in the ITU-T reference model [15] and as the IoMT edge network of the IoMT-based healthcare monitoring system. It consists of IoMT devices such as bio-sensors that keep track of a patient’s vital signs (e.g., heart rate, blood pressure, blood sugar), context-aware sensors for collecting context information from the user–patient environment (e.g., air pressure, humidity, sound, etc.), and actuators that support the provisioning of medical treatment (e.g., an insulin pump, which may be controlled remotely to inject the patient with insulin) in real time. In principle, the main goals of the perception domain are to connect objects (e.g., physical things) into the IoMT edge network and to measure, gather, and handle the information provided by these objects, through IoMT sensors, and then transmit the gathered information into the upper domain, via the Gateway, as shown in Figure 1.

The network domain is the transmission domain located at the layer above the perception domain and is deployed as the middle domain in the IoMT-based healthcare monitoring system architecture. The purpose of this domain is to receive the data, collected by the perception domain, and govern the transmission of the received data to the Cloud domain through integrated networks. This domain is an assemblage of various devices and communication technologies (e.g., Wi-Fi, 4G/5G, Internet).

Finally, the cloud domain is located on the upper layer of this architecture, and it receives the data from the network domain and leverages them so as to provide appropriate cloud-based services or operations to the user–patient, to healthcare professionals, or other authorized individuals (e.g., authorized relatives of the user–patient).

## 3. Blockchain Fundamentals: Consensus and Platforms

Consensus is a process of achieving agreement among nodes regarding the state of the data [16]. In this section, we present the most popular consensus mechanisms for blockchain-based IoT applications. In Table 1, we present a comparison of the three main consensus mechanisms. Afterwards, we present the main blockchain implementation platforms used in industry and academia.

### 3.1. Consensus Mechanisms

#### 3.1.1. Proof of Work

Proof of work (PoW) is a decentralized consensus mechanism for blockchain-based applications and the widest applicable blockchain consensus mechanism. Although PoW, as a distributed consensus mechanism, pre-existed as a primitive for synchronization between nodes, it possessed a limit in its scalability [17]. The first radical use of PoW consensus was introduced with Nakamoto’s Bitcoin Cryptocurrency in 2008 [18]. In short, PoW is a decentralized consensus mechanism in which the participants are required to prove their legitimacy by solving a mathematical puzzle which consumes time and energy.

Miners are the nodes of the network that, through repetition and failure of multiple processes, finally find a number known as nonce that satisfies specific criteria. For this number to be found, a certain amount of computational power needs to be used and consequentially a certain amount of energy (i.e., electricity). Once the nonce is calculated, it is appended to the hash of the block and the block is added to the chain. Miners function as guarantors of the integrity of the blocks and the blockchain provides a reward for their effort. This reward acts as a motivation for the miners to be part of the chain. Given the fact that the more miners that participate in the system, the easier the nonce can be found, many blockchains, mainly cryptocurrencies, apply another parameter to the equation—that of the mining difficulty. This keeps the block generation pace steady and prevents the possibility of tampering.

In short, the process of setting a transaction in the chain can be described in four steps: (i) at first the transactions are grouped together to form a block, (ii) the miners perform the PoW by solving the mathematical puzzle, (iii) reward is given to the miner that succeeds to solve the puzzle, (iv) the block is verified and committed to the chain.

For PoW to be tampered, more than 51% of the computational power of the network needs to be handled by a single entity in order to act maliciously and add a faulty block to the chain. This is not possible, especially in big chains, because the computational and energy cost to hold 51% of computational power is far more than the reward given by the tampering.

The PoW consensus is tamper-proof with high tolerance in malicious nodes and not susceptible to usual cybersecurity attacks (e.g., Sybil attacks, Man-in-the-Middle attacks), however, it comes with its limitations. PoW is not energy efficient and it cannot be used in applications where the nodes are resource-constrained (e.g., IoT). The mining equipment usually is by itself costly enough and due to the increase of computational power needed, it is possible to be led to a centralized protocol with potential security risks. Variants of this consensus protocols, among others, are:

- Proof of Capacity (PoC): in which instead of dependence on computation power, the mining process relies on hard disk capacity [19].
- Proof of Elapsed Time (PoET): is a consensus protocol proposed by Intel. The difference of this protocol with PoW is that the winning miner is selected randomly based on a random waiting time [19].
- Proof of Contribution: is a modification of PoW proposed in [20] by T. Xue et al. in order to increase the efficiency of miners.
- Kumar et al. at [21] proposed a variant of PoW based on statistical likelihood maximization and polynomial matrix factorization. This algorithm presents a significant reduction of memory and energy consumption from the devices as well as reduced convergence time. The authors propose this algorithm as a PoW algorithm suitable for resource constraint devices such as IoT nodes.

#### 3.1.2. Proof of Stake

Like PoW, Proof-of-Stake (PoS) is another basic consensus algorithm for blockchain. It solves issues that arise in the case of PoW, such as scalability, and block creation speed, and it is the second most popular consensus method for cryptocurrencies after PoW [19]. The PoS algorithm works similarly to PoW, but instead of computational power, the miner needs to provide some asset as a “stake” to participate in the mining process. The miner is selected by chance, but the possibilities to be selected are proportional to the amount of the “stake” it possesses. The miner uses a digital signature as a proof rather than solving a mathematical puzzle. The miner that validates the block is not awarded with a newly created asset but, with a transaction fee that the node that initiates, the transaction is obligated to pay. The malicious node is punished by losing the “stake” that it put in order to participate in the consensus process.

Therefore, PoS reduces the needed computational work on a blockchain in order to validate transactions and commit them in the blockchain. It reduces the scalability and energy sustainability issues that come with PoW consensus, and therefore PoS mechanisms produce an overall more sustainable blockchain.

In terms of security, this method is not vulnerable to the “51% attack” like in the case of PoW. To be able to control the chain, a node or a group of nodes needs to possess the 51% of the assets of the chain, which is very unlikely. However, in the case of PoS, there is a chance that a node does not possess enough assets so that in case it is selected as a miner and behaves maliciously, it has no assets to be deducted. This problem is called “nothing on stake”.

PoS is not a popular consensus algorithm for blockchain applications in IoMT edge networks. The main reason for this is that the IoMT edge networks, although they own assets (e.g., data), are not able to use these assets as an exchange means to be put at “stake” in order for the node to compete in a reward system. For this reason, its main use is in the case of cryptocurrencies, in which its low energy consumption is the main reason for the choice.

Another variant of this consensus method is the Delegated Proof of Stake (DPoS), which uses a representative democratic method based on the stakeholder, in comparison with the direct democratic PoS [19]. Finally, some other, less popular, in terms of application, mechanisms based on PoS include the following [19]: Leased Proof of Stake (LpoS), Proof of Importance (PoI), and Proof of Burn (PoB).

#### 3.1.3. Byzantine Fault Tolerance

The following protocols are based on the Byzantine generals’ problem, in which a number of parties try to achieve consensus without trusting each other fully and they can only send a message to one another, without knowing which of the parties is malicious or faulty.

Practical Byzantine Fault Tolerance (PBFT) is a Byzantine Fault Tolerance algorithm. It is a consensus algorithm that is used basically in permissioned blockchain networks. The main function of this algorithm lies on three phases of message exchange in order agreement to be achieved [22]. The three phases are pre-prepare, prepare, and commit.

**Figure 2:**
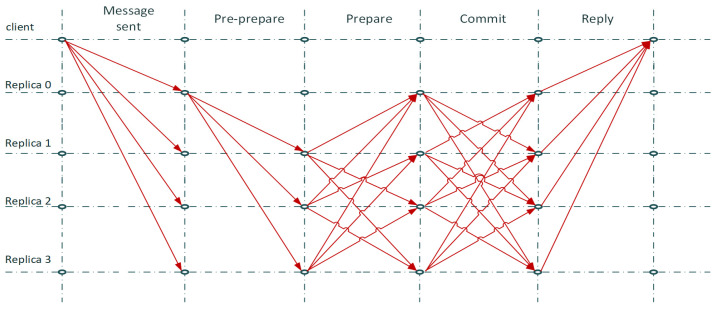
PBFT diagram.

The client node sends a message to a primary node, namely replica 0, which will broadcast it to all the other nodes and replicas. It can be noted that the replica 0, or primary node, is changed in each consensus round and it can be substituted by a view change protocol, meaning that each node of the system has a potential to be a replica 0 node. The replica 0 node assigns metadata and certificates to the messages that are sent and checked by the other replica nodes. This is the pre-prepare phase. The prepare phase follows, in which all the replica nodes multicast back the message to the other replica nodes, by adding another certificate to it. In the case that the pre-prepare and prepare certificates of the messages received by the replica match, then the replica will multicast a commit message. After the commit message is received, the replicas execute the request of the client and broadcast back a reply [23]. PBFT uses symmetric cryptography (i.e., MAC) instead of public key signatures for message authentication. In Figure 2, we present a diagram of the PBFT algorithm.

The security condition of the PBFT algorithm is that the consensus has to be agreed among more than two thirds of the nodes. This means that a network with more than one third faulty or malicious nodes cannot function securely and could be compromised. This leads to the conclusion that as the number of nodes of a system increases, the more secure the system becomes. In this case, we can mark the limitation of this algorithm as its susceptibility to Sybil attacks, and the excessive communication overhead that can be witnessed in the case of an increased number of nodes, which leads to scalability issues. However, it is a lightweight solution for permissioned blockchains in networks such as IoMT edge networks, because it has high throughput, low latency, and low computational overhead [19,24]. Other variations of BFT algorithms include the following:

- Delegated Byzantine Fault Tolerance (dBFT): this is practically the same as PBFT, but in this case not all the nodes are necessary to participate in order to achieve consensus [19].
- Stellar: it is used for micro finance services and uses federated Byzantine Fault Tolerance (FBFT) [25].
- Ripple: similar to Stellar [26].
- Tendermint: it is a BFT consensus protocol in which the nodes have different voting power in relation to their stakes [27].

### 3.2. Blockchain Platforms

Blockchain platforms are environments in which blockchain-based applications can be deployed. In this section, we present the main blockchain implementation platforms used in industry and academia.

Ethereum is a public-permissionless blockchain framework [28]. Although Ethereum’s consensus protocol is PoW, there is also a version of Ethereum named Casper which functions with PoS consensus protocol. Ethereum is a highly decentralized blockchain implementation platform with high scalability. However, due to PoW consensus, it has low transaction throughput. With regard to smart contract deployment, it is possible with Solidity [29], a contract-oriented high level language.

Hyperledger fabric has been proposed by Androulaki et al. [30], and it is a distributed ledger platform for developing applications with modular architecture [31]. This platform provides pluggable consensus protocols (mainly PBFT) and a private-permissioned blockchain model. It is suitable for deploying IoT applications for stakeholders that partially trust each other. This implementation platform has low scalability due to the nature of PBFT algorithms and 33.33% (1/3) adversary tolerance. However, it provides high privacy and throughput and supports the development of smart contracts.

Hyperledger Sawtooth is an enterprise solution for blockchain deployment [32]. Its consensus protocol is Proof of Elapsed Time, and it is a permissioned and private blockchain. In comparison with Hyperledger Fabric, it is superior in terms of scalability, because of its more scalable consensus algorithm. Nevertheless, its adversary tolerance is not verified.

Bitcoin is suitable mostly for applications regarding digital currencies and transactions [33]. The transactions are verified with the PoW consensus mechanism and it constitutes a public and permissionless blockchain. Its nature permits high decentralization, scalability, and high adversary tolerance, but due to the amount of time taken to verify transactions it has a low throughput in comparison with other blockchain implementation platforms. Bitcoin Blockchain functions with the use of the cryptocurrency Bitcoin (BTC) as a reward for the nodes that participate in the mining process. Given the fact that this is the earliest deployed platform, its smart contract deployment capability is limited.

Last but not least, other blockchain development platforms include Corda, a permissioned blockchain with Pluggable consensus protocol [34], and Iota, a public-permissionless blockchain with Tangle consensus mechanism [35].

## 4. Blockchain-Based Security for IoMT Edge Networks

In this section, we examine the following two types of blockchain-based security mechanisms: (a) the very few existing ones specifically designed for IoMT edge networks, and (b) those designed for other types of IoT edge networks but could be possibly adopted in IoMT edge networks due to similar capabilities and technical characteristics between other types of IoT edge networks and IoMT edge networks. In particular, our target is to provide a foundation for organizing research efforts towards the design and development of reliable blockchain-based blockchain-based security mechanisms, ensuring authentication and authorization as well as implementing AIDSs for IoT edge networks.

### 4.1. Blockchain-Based Authentication for IoMT Edge Networks

In this section, we present some existing works on blockchain-based authentication mechanisms for IoMT edge networks and a number of works related to blockchain-based authentication mechanisms applied to other types of IoT networks that, however, could be possibly adopted in IoMT edge networks.

#### 4.1.1. Existing Blockchain-Based Authentication Mechanisms for IoMT Edge Networks

R. Akkaoui in [36] proposes a scalable authentication scheme for Internet of Medical Things (IoMT) devices based on smart-contracts, leveraging the physical unclonable function (PUF) as an additional authentication factor. PUF is a random unique device identifier based on the physical characteristics of an electronic circuit. The proposed scheme is designed for authentication and firmware update purposes. The authentication scheme is named smart contract against counterfeit IoMT (SCACIoMT). The certificate generation is possible with the use of ECC, and the scheme is implemented in the Ethereum platform. In the context of this survey, we are going to focus on the authentication approach.

The architecture of the proposed scheme consists of the following entities: the authorized nodes, which are semi-trusted nodes responsible for mining transactions, with proof of authority (PoA) consensus and block creation. The manufacturer nodes are authorized nodes. They do not perform block creation, but they are responsible for updating the authenticated devices lists. The patients are the final category of entities in the system, which are the data generators, i.e., the entities that create data and forward them to the blockchain as transactions to be sealed into blocks.

**Figure 3:**
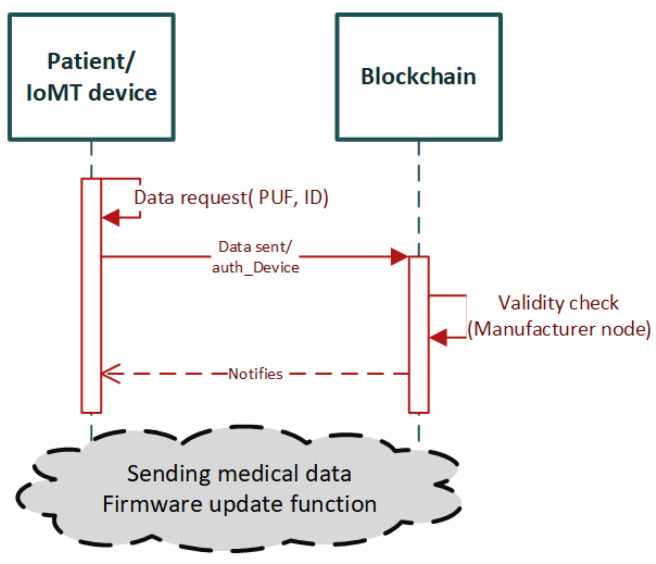
Sequence diagram summarizing the workflow of the proposed scheme in [36].

The authors present a detailed workflow of the architecture; however, a brief description of the authentication scheme is presented in the sequence diagram in Figure 3 The device recovers its properties (i.e., ID, PUF, hash firmware) and sends the data together with the Ethereum address as a transaction to the blockchain initiating the authentication. A manufacturer node then initiates a data verification transaction to validate the provided data. Once the data are validated, the manufacturer node notifies the patient and the patient is able to provide medical data. The authorised nodes update the blockchain accordingly as the final step of the authorisation process. The transactions are held by a series of smart contracts.

The author has provided a detailed security and performance analysis regarding the implemented scheme in terms of privacy and confidentiality. The scheme is implemented on a privately built Ethereum-based blockchain using the Geth client, running on an Ubuntu virtual machine v 14.04.6 [36] with the following host machine specifications: Intel i3-3110M, 2.4-GHz, 4-GB 1600-MHz DDR3 [36]. The scheme is evaluated regarding computational cost, communication, and storage cost.

Overall, the present research work provides a detailed implementation methodology and evaluation results, as well as a complete design of the scheme and the algorithms of smart contracts, written in Solidity programming language. Moreover, it provides solid solutions to security issues such as data privacy and information high jacking, and eliminates the single point of failure with a decentralized architecture. On top of that, the user’s credibility is important, and it is also taken into consideration as a factor for the system to function properly. To the best of our knowledge, the proposed scheme could be considered as the most complete blockchain-based scheme for IoMT edge networks.

Fotopoulos et al. in [37] proposed a novel IoMT authentication mechanism for patient data collection, process, and storing in a healthcare environment. They include approaches such as self-sovereign identity (SSI), zero knowledge proof, and blockchain to create a decentralized mechanism for effective authentication of medical devices. The novelty of this research work is based in the inclusion of SSI as a new technology, which provides inherent protection from impersonation attacks and provides robust integrity and data privacy, in connection with blockchain technology. The proposed mechanism is specifically designed for device authentication in IoMT edge network, and it is considered suitable for healthcare use cases. Although authors have proposed Hyperledger fabric as an implementation platform for their proposed mechanism, they will prepare the implementation of their blockchain-based mechanism for real case scenarios as future work.

#### 4.1.2. Potential Blockchain-Based Authentication Mechanisms for IoMT Edge Networks

D. Li et al. [38] proposed a blockchain-based authentication mechanism for IoT in order to eliminate the single point of failure. In their proposed research, they point out the necessity of device authentication without the use of a central authority, which is used in the traditional Public Key Infrastructure mechanisms (PKI). Blockchain technology is suitable in this architecture and provides the decentralized network structure.

The system model of the proposed architecture consists of a multi-node network and focuses on device registration and storing the hash of each device’s information (i.e., ID, public key) in the blockchain ledger. The hashing of this information also provides the benefit of data integrity, as alterations in data can be detected through it. The system operates in the following functions: the enrolment of devices, the identity authentication, and the integrity verification. Nodes of the network function either as consensus nodes which take part in the consensus process, or non-consensus nodes that are used only for data transferring. The role of each node is defined by the needs of the permissioned chain.

The enrolment process is initiated when a certain device communicates a connection request to the network. For a device to be enrolled, a key pair is generated, from which the private key is encrypted and stored in a local storage while the public key is stored in the blockchain ledger. After the consensus process takes place in the consensus nodes, with the use of Practical Byzantine Fault Tolerance (PBFT) algorithm, a block is generated and propagated to all the nodes of the network (i.e., consensus, non-consensus). The identity authentication takes place under a P2P authentication method.

**Figure 4:**
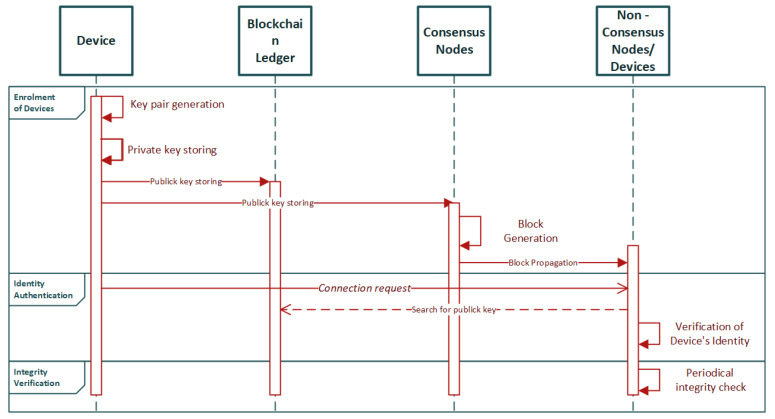
Sequence diagram of the proposed scheme in [38].

Integrity check of data is also possible through the proposed mechanism. It is accomplished by nodes that periodically communicate a request for integrity verification to their neighboring nodes. In the case of blockchain-based mechanisms, integrity checks are based on the use of hash encryption techniques rather than traditional methods of asymmetric key encryption. The three system operations are depicted in the sequence diagram in Figure 4.

The authors have moved forward in the implementation of the blockchain-based authentication mechanism with the use of Raspberry Pi devices and the Hyperledger Fabric platform for system deployment. Hyperledger Fabric’s nature permits the creation of multiple channels in an ad-hoc network of IoT nodes, and each node can communicate through each of these channels—if permitted—without interference. As a result of the connection of the nodes through a blockchain network, the interaction between them takes place in the form of transactions that occur inside the network. These transactions are device enrolment, identity authentication, and integrity check. To generate the keys, the authors use a cryptographical secure pseudo-random number generator (CSPRNG) that ensures the randomness of the generated key. The stimulation of the CSPRNG originates from information collected from IoT devices such as CPU clock, number of processes, etc.

The proposed research constitutes a complete work with a generic design that can be applicable in many use case scenarios and can be adapted to other specific architectures. It takes advantage of the decentralized nature of the blockchain and the permissioned aspect of Hyperledger Fabric to create a solid design that overcomes the drawbacks of traditional authentication mechanisms, and it is lightweight in implementation which makes it suitable for IoMT edge networks.

Authors in [39] propose a blockchain-based distributed authentication mechanism to allow communication among devices from various IoT systems. The system architecture is separated into two layers: Device layer and Fog layer. Device layer contains the IoT systems that themselves contain the smart devices, while the Fog layer contains the blockchain network nodes, which are by definition legitimate and trusted. The proposed architecture provides three types of communication: (i) device-to-fog communication, meant for device registration and authentication, (ii) fog-to-fog communication, meant for synchronization of the authentication data with all the blockchain nodes, and (iii) device-to-device communication, which permits the communication between two already authenticated devices. The proposed mechanism also provides access control, but in the context of this survey we will focus on the authentication part.

**Figure 5:**
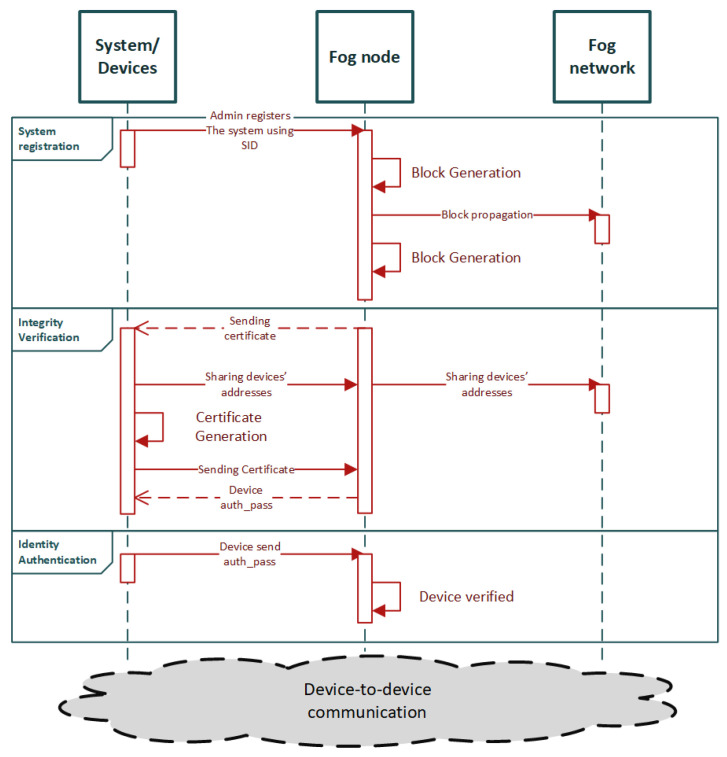
Sequence diagram of the proposed scheme in [39].

The authentication process in the proposed mechanism takes place as follows: blockchain nodes (i.e., located at the Fog layer) are connected to one or many IoT systems of the Device layer. Each IoT system chooses an adjacent blockchain node and the registration is taking place between the IoT system and the corresponding node. The system is registered by using a unique System ID (SID), which is generated by the admin of the system. This SID is provided and validated by the blockchain node. Then the SID, if valid, is stored as a transaction in the blockchain, and correspondingly the blocks are propagated to the other blockchain nodes. After the end of the system registration phase, the system admin is provided with a certificate by the blockchain node, which is sent as a transaction in order to proceed to the device registration. For the generation of the certificate, a private key is used. Then, the device registration phase takes place through a similar process with the generation of registration-token certificates. The device authentication is the last phase of this process, where the already registered devices need to be authenticated through the blockchain network. After the authentication process is completed, the blockchain network is used for device communication between systems. The authentication process is presented in a sequence diagram in Figure 5.

The proposed mechanism comprises a complete and thorough research work. It is evaluated according to the security requirements and against attacks, as well as in terms of execution time and power consumption. It complies with the necessary security requirements such as integrity, non-repudiation, authentication, and regular attack types that may occur in an IoT network. The implementation is done with the aid of Ethereum Blockchain, which is suitable for the implementation of the system. The evaluation provides results, in terms of execution time and power consumption, demonstrating better performance in comparison with the already established state-of-the-art techniques. In addition, the proposed mechanism provides better scalability capabilities in terms of number of devices and transactions per time unit. Although the proposed authentication approach targets general purpose IoT networks, it could also be a fitting solution for IoMT edge networks due to its lightweight characteristics.

M. T. Hammi et al. at [40] have proposed a decentralized system for device identification and authentication named bubbles of trust. In their proposed work, they divide the network devices into groups, or zones as it is referred to in the manuscript, that communicate with each other. These zones are named as bubbles and the communication inside the zones takes place as blockchain transactions. The architecture is based on a public blockchain, so it is easier for new users to register.

**Figure 6:**
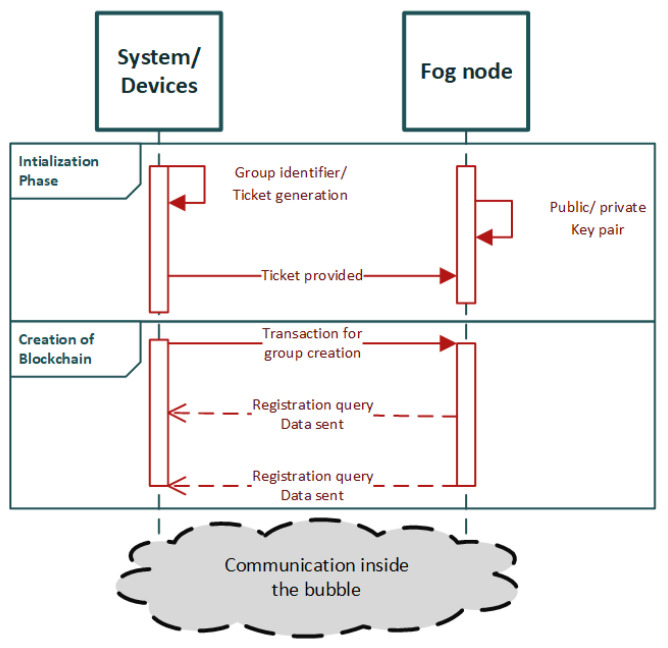
Sequence diagram of bubbles of trust [40].

The lifecycle of the architecture begins with the initialization phase. A device is predefined as a Master device. The rest of the devices that participate in the network are Follower Devices. The Master device creates the group identifier and generates tickets—a certificate equivalent—to be provided to Follower devices so they can be enrolled in the system. The ticket contains information regarding the group and data of the Follower device to be enrolled. The Follower devices generate a private/public key pair themselves with Elliptic Curve Cryptography (ECC). After the initialization phase, the lifecycle continues to the creation of the zone in blockchain level. In this phase, the Master initiates a transaction in order to create the group of devices that will take part in the blockchain. Afterwards, the Follower devices initiate transactions in order to enroll into the blockchain, whose identities are checked through a smart contract, and if they are valid then the devices are added to the network. Then, no further authentication process is needed, and the Follower devices are part of the blockchain group where they can communicate inside the bubble through a series of smart contracts that are also validated in the blockchain. The process is depicted in the sequence diagram in Figure 6.

Authors have preceded the implementation of the architecture with the use of two HP laptops (OS Ubuntu 14.04) [40] and 1 Raspberry Pi (OS Raspbian 4.9.41) [40] as end nodes and the use of Ethereum blockchain as a framework. The interaction between the nodes takes place through Ethereum smart contracts written in Solidity language. Regarding the interaction between the end-nodes and the blockchain, authors have implemented a C++ interface that encodes/decodes data toward/from Ethereum. The implementation is performed in the context of the evaluation of the proposed architecture where the evaluation results present considerable durability against IoT network attacks (e.g., DDoS attacks, message replay, spoofing attacks, sybil attacks). In addition, satisfactory results in terms of time and energy consumption, as well as financial costs, were demonstrated.

The authors refer to a future work to: (i) evolve the system to allow controlled communication between a chosen set of bubble, (ii) proceed with the implementation of the architecture, and (iii) design a protocol that optimizes the number of the miners that will work in the system. As open issues, they note the fact that the proposed architecture is not adapted to real time applications, has not had an initialization phase—something that was provided in the other related works [38,39] —and is dependent on the evolution of cryptocurrency rate. Overall, it comprises a complete research work for a blockchain-based authentication mechanism and promises to be applicable to different use cases (e.g., smart house, smart factory, waste management). Furthermore, it is worthwhile mentioning that the design characteristics and evaluation results of the proposed system make it a potential authentication solution to enhance security in IoMT edge networks for healthcare monitoring systems.

M. Zhaofeng et al. propose a decentralized blockchain-based authentication scheme in [41] named BlockAuth. In this scheme, each device on the IoT edge layer is considered as a blockchain network node, which acts as a participant in the blockchain. The scheme is fault-reliable and decentralized to eliminate the single point of failure, and also suitable for identity authentication technologies (e.g., password-based, certificate-based, biotechnology-based). The registration phase is covered by a certificate authority that issues the certificates and it is initiated by a user registration request, which is sent as a transaction through a smart contract. Then, in the authentication phase, the identity and the transaction hash are checked by the blockchain in order to verify the identity of the user. The consensus mechanism used in this scheme is a PBFT algorithm.

The authors move forward to the evaluation of the scheme through a virtual machine environment by implementing the registration, authentication, and consensus algorithms in order to compare the BlockAuth scheme with already implemented related schemes. The results are satisfying with BlochAuth providing advantages in terms of multi-signature identity data, big fault tolerance, and strong security and reliability. On the other hand, the time complexity of the proposed scheme is higher than the others it is compared with. However, despite its high time complexity, the proposed scheme could still be considered as a solution for IoMT edge networks in healthcare monitoring systems, given its decentralized nature, low power consumption, and strong security features.

Authors in [42] propose a mutual authentication scheme based on blockchain solutions for IoT network, and, specifically, for the use case of smart home, named HomeChain. They integrate blockchain and group signature to provide anonymous authentication inside the IoT network. The proposed scheme uses public key encryption for the generation and distribution of the keys with the use of an ECC algorithm scheme. The group signatures are used for the signing of the transactions that are held inside the blockchain. The proposed scheme uses a permissioned blockchain, and Practical Byzantine Fault Tolerance (PBFT), as a consensus algorithm. The system includes:

- a user that owns a group of IoT/smart devices functioning as a user node
- a blockchain network with consensus nodes, and
- a smart home network that consists of: (i) a group of smart devices, and (ii) a gateway that connects the smart house to the blockchain network.

The implementation of the system has been held on JUICE (an opened permissioned blockchain service platform) and the evaluation results show reliability on various IoT network attacks (e.g., Man-in-the-Middle Attack, Replay attack, DDoS attack) and sufficient performance in terms of time consumption in comparison with other related works. This research work proposes a complete blockchain-based authentication scheme with implementation and promising results. On top of that, the authors consider the attribute-based cryptographic approach in order to achieve better access control. In principle, its design characteristics and evaluation results make it a potential authentication mechanism to enhance security in IoMT edge networks for healthcare monitoring systems.

Q. Fan et al. in [43] propose an ID-based signature authentication and secure data sharing scheme for IoT. The scheme has been deployed in a three-layer IoT model (i.e., Perception Layer, Network Layer and Application Layer) with the use of a blockchain layer that is also divided into two sublayers—consensus layer and propagation layer. The authors present detailed algorithms for the different phases of the authentication and the key generation. The scheme has been evaluated and meet the security requirements of an IoT network as well as being resilient to common IoT network attacks and threats. Although the research work provides improved and satisfactory result in the evaluation of the scheme, a particular blockchain platform has not been mentioned to be used for this evaluation. The present research work is in its early stages, since a specific blockchain platform has not been used for the mechanisms to be implemented, however, it is a promising scheme that could be adapted to a healthcare monitoring system, relying on an IoMT edge network.

Authors in [44] present a blockchain-based multi-WSN (wireless sensor network) authentication scheme for IoT with the use of a private blockchain. To establish the scheme, notable assumptions have been made that can easily be verified:

- each IoT node has a unique Ethernet address
- cluster head nodes and base stations have certain storage and computing capabilities, and smart contract can be deployed
- as a node manager in a single network, base station is trusted by the nodes in the network; and
- the process of initialization of the nodes is secure.

The network model consists of base stations, cluster head nodes, ordinary nodes, and the end user. The authentication working cycle includes an initialization phase and the authors have proceeded into the implementation of the scheme. The scheme provides novelty in terms of the hierarchical multi-WSN network design, the hybrid model of the blockchain, and a mutual authentication scheme for IoT nodes that enhances the scalability of the IoT authentication. The evaluation proves durability of the scheme against IoT network attacks and meets the security requirements of such schemes. Therefore, it could comprise an option for ensuring blockchain-based authentication in IoMT edge networks.

For each blockchain-based authentication mechanism presented in this section, Table 2 summarizes their advantages and limitations, the type of blockchain and the blockchain platform used, implementation parameters, and planned work.

### 4.2. Blockchain-Based Authorization for IoMT Edge Networks

In this section, we present works related to blockchain-based authorization mechanisms applied to other types of IoT networks that, however, could be possibly adopted in IoMT edge networks. It is worthwhile mentioning that, to the best of our knowledge, blockchain-based authorization mechanisms specifically designed for IoMT edge networks do not exist in the literature.

**Figure 7:**
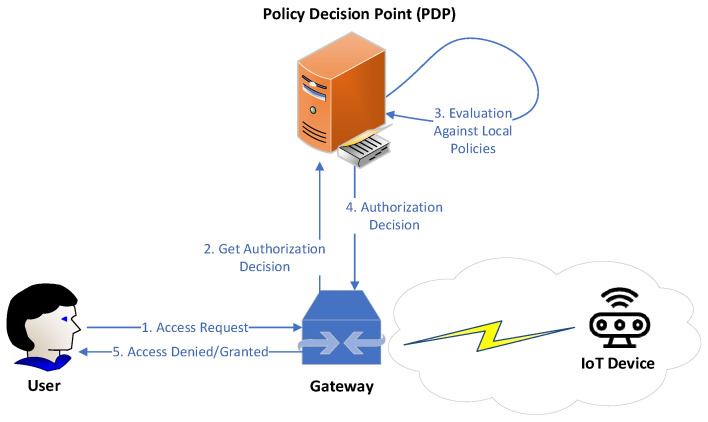
Centralized Approach [48].

**Figure 8:**
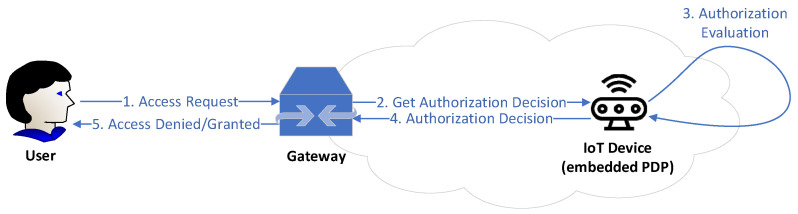
Distributed Approach [48].

Ronghua Xu et al. [45] highlighted that access authorization comprise one of the top security and privacy challenges that IoT has to address for its wide adoption in order to ensure secure resource and information sharing. They discuss that the centralized authorization server of traditional access control (AC) may be the single point of failure or the performance bottleneck. Towards this direction, the authors designed and developed a prototype of a capability-based decentralized mechanism (BlendCAC) using Blockchain technology which uses token management for various actions (e.g., permissions or revocations on access authorization) making the authorization decision. Capability-based access control is an access control model commonly used in the distributed architectures, where the access control logic is embedded and distributed into the end devices and not into a central authority [45,46,47,48]. These devices, also referred to as “smart things” or “smart objects” [48], are being enabled with capabilities that make them able to obtain, process, and send information about the access control rights of the entities of the system to other entities and/or services [48]. Thereby, the “smart things” are able to carry out the authorization process, without requiring a central authority (see in Figure 7 and Figure 8 an example with a motion sensor). In this context, we suppose that capability-based access control constitutes a suitable access control solution to enhance the authorization of IoMT edge networks in IoMT-based healthcare monitoring systems, distributing the access control logic across the increasing number of resource-constrained medical devices connected to IoMT edge networks.

**Figure 9:**
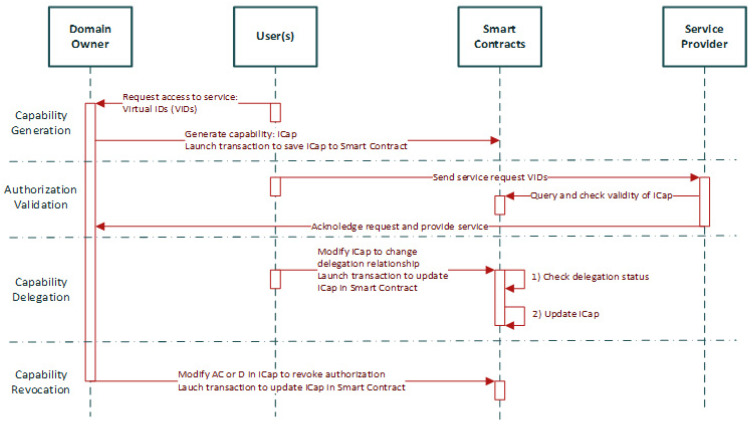
BlendCAC Block Diagram.

**Figure 10:**
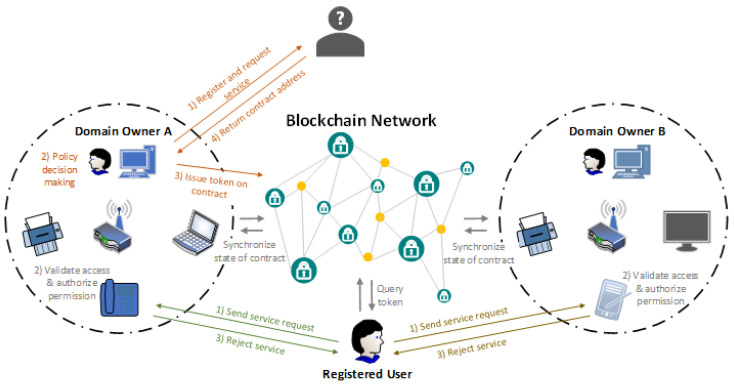
BlendCAC System Architecture.

The aim of the proposed BLockchain-ENabled Decentralized Capability-based Access Control or BlendCAC, is the facilitation of effective access control processes for devices, services, and information in large-scale IoT systems [45]. Based on the blockchain network, the authors propose a capability delegation mechanism for access permission propagation, whose main functions are as illustrated in Figure 9. On top of that, the mechanism takes advantage of a smart contract for registration, propagation, and revocation of the access authorization, creating a robust identity-based capability token management strategy. In the proposed BlendCAC scheme, IoT devices are not overseen by a centralized authority. On the contrary, they are their own master to control their resources, which is the main idea of capability-based systems. In Figure 10, the proposed BlendCAC system architecture is illustrated under the use case scenario of two isolated IoT-based service domains without pre-establishing a trust relationship between them. Each domain has a domain owner which has the ownership of several IoT devices, and thus it is able to enforce predefined security policies to manage all the domain related devices and subsequent services. At this point, it is important to observe that, essentially, every domain involves a domain owner which, after all, is a centralized entity; this might cause issues such as single point of failure, bottleneck, performance degradation, etc. similarly to centralized approaches. Finally, every domain owner maintains a local chain with the transactions that happened in their domain, which then must be periodically synchronized with the global Blockchain.

The authors implemented and tested their proposed scheme on a local private blockchain network, using devices such as Raspberry Pi and laptops/desktops. Their experimental results showed the feasibility of BlendCAC to offer a lightweight, scalable, decentralized, and fine-grained access control solution for large-scale IoT systems.

**Figure 11:**
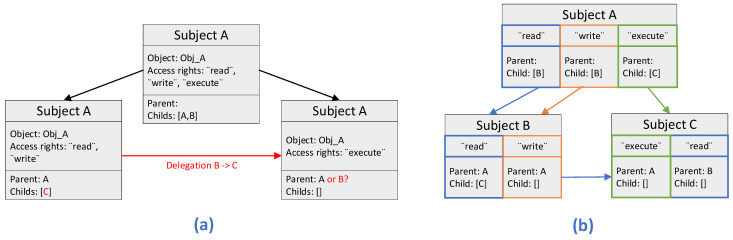
(**a**) Delegation tree, and (**b**) Delegation graph.

In [47], the authors examined the BlendCAC scheme [48], identified its limitations, and tried to address them. In particular, they pointed out that in BlendCAC, a subject cannot obtain rights from more than one subject. This is because the BlendCAC scheme manages the capabilities of subjects and their delegation relationships with each object by using a delegation tree. In this context, if we consider that subject A is the parent of subjects B and C (in accordance with the delegation tree structure as illustrated in Figure 11a), then subject A can give access rights to subjects B and C for the objects that belong to subject A. However, subject B, as it is not actually the parent of subject C, is not able to give any access rights to subject C for the objects that belong to subject B. In addition, to complete a delegation, the related tokens, namely ICap and IDC tokens, must be updated synchronously. This requirement is not always feasible to be fulfilled in the blockchain system, taking into consideration the difference of the times when the two transactions for updating the tokens are included into the blockchain.

**Figure 12:**
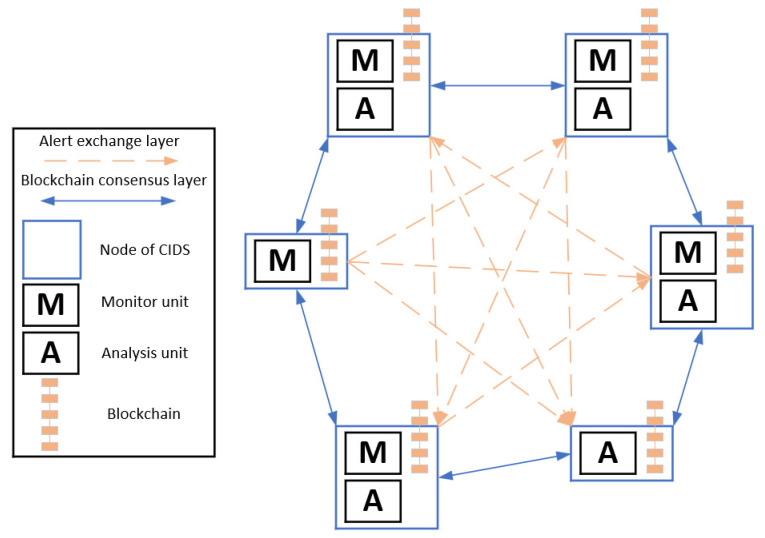
Architecture for the incorporation of blockchain into a CIDS.

Therefore, the authors in [47] proposed a novel smart contract-based CapBAC scheme enabled with more flexible capability delegation and more fine-grained capability management in order to deal with the limitations of the BlendCAC scheme that we have mentioned previously. More specifically, the authors firstly define the capability tokens in units of authorized actions. In this way, they achieve having one token per action rather than one token per subject, as it is in the BlendCAC scheme. To address the second limitation, the authors introduce the usage of one single type of token to summarize the information of capabilities and delegation relationship so as to be feasible to update this information simultaneously when needed. On top of that, to enable more flexible capability delegation, the authors manage the delegation relationship of the different subjects by a delegation graph as opposed to the delegation tree introduced in the BlendCAC scheme (see Figure 12). Their novel proposed scheme also supports the functionality of adding new authorized actions, which is not possible in the BlendCAC scheme.

Overall, the authors in [47] propose a Capability-Based Access Control (CapBAC) scheme by applying the emerging Ethereum blockchain technology. Their scheme makes use of Ethereum smart contracts (i.e., executable codes residing in the blockchain) to store and manage the capability tokens (i.e., special data structures that define the permitted actions of a user, also referred to as subject, on a certain resource, and also referred to as object). Their scheme provides more fine-grained access control and more flexible token management, defining capability tokens in units of actions. On top of that, for storing the token delegation relationship among the different subjects, they deploy a delegation graph. Most of the existing smart contract-based CapBAC schemes use the delegation tree, including the BlendCAC. Their scheme enables object owners with the capability to verify the ownership and validity of the capability tokens of the subjects by storing the tokens and the delegation graph in smart contracts. Finally, the authors constructed a local Ethereum blockchain network and conducted extensive experiments demonstrating the feasibility of the proposed scheme large-scale and trustless nature of the Internet of Things (IoT), showing promising results for its deployment in IoT-based Healthcare applications. In this regard, this Capability-Based Access Control (CapBAC) scheme shows potential applicability in the IoMT edge networks.

In [49], the authors combine the blockchain smart contract technology and the attribute-based access control (ABAC) model and propose a novel distributed and reliable access control framework for smart cities. It is important to highlight that ABAC refers “to an access control approach in which access is mediated based on attributes associated with subjects (requesters) and the objects to be accessed” [50]. In particular, each object and subject are associated with a set of attributes, such as time of creation, location, access rights, etc., and the access to an object is then authorized/denied depending upon whether the required (e.g., policy-defined) correlation can be made between the attributes of that particular object and of the requesting subject.

The proposed framework consists of:

1. a Policy Management Contract (PMC) that is responsible for managing the ABAC policies
2. a Subject Attribute Management Contract (SAMC) that is responsible for managing the attributes of subjects (i.e., entities gaining access to resources/objects)
3. an Object Attribute Management Contract (OAMC) that is responsible for managing the attributes of objects (i.e., resources being accessed), and
4. an Access Control Contract (ACC) that is responsible for performing the access control.

The authors construct a local private Ethereum blockchain system in order to deploy the four smart contracts, conduct extensive experiments to evaluate the monetary cost and, finally, compare the performance evaluation of the proposed framework with existing access control list (ACL)-based scheme in the literature review. The experimental results showed feasibility of the integration of the proposed framework in large-scale IoT environments, making it a promising potential solution for the IoMT edge networks in IoMT-based healthcare monitoring systems. Although the proposed framework introduces a larger deployment cost at the deployment stage, compared to other ACL-based schemes, it introduces less monetary cost during the system running, especially for large-scale IoT systems consisting of a large number of subjects and objects with common attributes. Smart cities comprise a typical example of such systems. However, although the prototype demonstrates the feasibility of the proposed framework, it can hardly reflect the performance of the framework in large-scale IoT applications such as smart manufacturing or healthcare. The authors will consider, as future work, the implementation of the proposed framework in larger-scale environments.

Apart from the monetary cost, another major concern of the proposed ABAC framework in [49] is the throughput issue. In particular, this concern refers to the total number of access requests that can be processed per unit time (e.g., second). The throughput of the proposed framework depends greatly on the throughput of the underlying blockchain systems (i.e., number of transactions included in the blockchain per second). In their implementation, the authors deployed Ethereum 1.0 as the underlying blockchain system, the throughput of which is about 15 transactions per second [51]. Additionally, further latency is introduced to the access control process, reducing the throughput of the framework since the ACC (i.e., access request processing unit) needs to communicate with other contracts through messages. Actually, the consensus algorithm is one of the main reasons for the throughput being low. Their implementation is based on the widely used Proof-of-Work (PoW) algorithm, which involves a vast number of calculations to add one block of transactions into the blockchain. The authors also highlight that Ethereum 2.0 comprises a promising solution, which changes the consensus algorithm from PoW to Proof of Stake (PoS) and adopts the method of sharing to greatly enhance the throughput performance [52]. It is expected that Ethereum 2.0 will enable 64 to several hundred times more throughput than Ethereum 1.0. Therefore, the author’s future work is to implement their proposed ABAC framework on Ethereum 2.0 to significantly improve its throughput performance. Table 3 summarizes our findings on blockchain -based authorization mechanisms which have been designed for other types of IoT networks but could be possibly adopted in IoMT edge networks presenting their main guarantees and drawbacks.

### 4.3. Blockchain-Based Intrusion Detection Systems (IDS) for IoMT Edge Networks

In this section, we present works related to blockchain-based IDSs applied to other types of IoT networks that, however, could be possibly adopted in IoMT edge networks. It is worthwhile mentioning that, to the best of our knowledge, blockchain-based IDSs specifically designed for IoMT edge networks do not exist in the literature.

Alexopoulos et al. [54] proposed the idea of leveraging blockchain technology in or-der to enhance the operations of Collaborative Intrusion Detection Systems (CIDSs). Blockchain could enhance the trust between the nodes of a CIDS, provide a means to ensure accountability, and offer an appropriate consensus mechanism. The authors described a set of requirements for an effective and trustworthy CIDS, which are presented in Table 4.

Figure 12 demonstrates the general architecture proposed by the authors in [54] for the incorporation of the blockchain technology into a CIDS. Each node in Figure 12 represents an intrusion detection system (IDS). The participating nodes perform monitoring operations or analysis operations or both types of operations at the same time. The raw alert data produced during the monitoring operations can be considered as transactions, and thus can be stored in a blockchain that is replicated among all the participating nodes of the CIDS. A new transaction (i.e., alert data) should be included in the blockchain only after the participating nodes have run a consensus protocol that verifies the validity of the transaction. As a result, only well-formed alert data are contained in the blockchain ledger, and the integrity and tamper-resistance of the alert data is guaranteed. At the same time, the alert data are visible by all of the participating entities and therefore, accountability is ensured. In addition, the use of a secure distributed ledger (i.e., blockchain) eliminates the existence of single-points-of-failures (SPoFs), and thus the system is more resilient. Moreover, the authors in [54] suggest that the communication overhead can be reduced by storing hashes of the alert data in the blockchain instead of raw alert data.

As shown in Figure 12, the communication between the nodes is divided in two logical layers, namely the Alert Exchange layer and the Consensus layer. The Alert Exchange layer is responsible for disseminating the alert data to the participating nodes. In particular, each participating node transmits and/or collects alert data based on its role as monitor and/or analysis unit, while the specific communication mechanism (e.g., flooding or gossiping protocol or on-demand data exchange) depends on the needs of the CIDS. On the other hand, the Consensus layer is responsible for the verification of the transactions and their inclusion to the ledger.

The description of the system architecture concludes by exploring possible cases where specific alert data need to be kept confidential among a subset of participating nodes. To address this case, the authors suggested that a separate blockchain containing the confidential alert data should be created by the nodes that should have access to these alert data. Furthermore, encryption could be employed in both layers for the alert data. Thus, the nodes, which do not hold the specific secret key, cannot access the alert data exchanged and processed among the certified participants. Finally, the authors discussed several design considerations, along with their corresponding implementation options. The implementation options for each design consideration, as described by the authors in [54], are summarized in Table 5.

The authors in [54] provide a general concept of blockchain-based CIDSs without referring to a specific type of network. However, it is possible to adapt the proposed concept in IoMT edge networks if we consider that the participating nodes are IoMT gateways. In this case, the gateways are the devices that will form a CIDS in order to safeguard themselves, their connected IoMT devices (e.g., IoMT sensors and/or actuators), and the IoMT edge network.

Golomb et al. at [57] proposed a lightweight framework named CIoTA, which utilizes the blockchain technology so that distributed and collaborative anomaly detection can be performed on IoT devices, such as Raspberry Pi. Initially, the authors in [57] assumed a network of multiple interconnected IoT devices (i.e., Raspberry Pi devices), where an anomaly-based intrusion detection system (AIDS) is installed on each IoT device.

Then, the authors elaborate that adversaries could potentially exploit the AIDS of an IoT device during its training phase. During this phase, the AIDS train an anomaly detection model to capture the device’s normal behavior and all the observations are considered as benign. Thus, the interference of an adversary during this phase can negatively impact the detection accuracy. In addition, the authors assume that in the case that all the IoT devices of the same type could simultaneously begin training their own anomaly detection model based on their own locally observed behaviors, it becomes unlikely that the majority of these IoT devices could be exploited before completing their training phases. Based on this assumption, the proposed CIoTA framework employs the blockchain technology in order to perform distributed and collaborative anomaly detection on IoT devices.

In a network of interconnected IoT devices, each IoT device possesses an agent which creates and maintains a local anomaly detection model. In particular, a local extensible Markov model (EMM) is trained according to the regular memory jump sequences of programs running on the IoT device. The EMM can be updated and merged with other EMMs. This ability of the EMM is leveraged by the CIoTA framework, which uses the blockchain in order to merge the locally trained anomaly detection models (i.e., EMMs) and incrementally produce a trusted anomaly detection model via self-attestation and consensus among the connected IoT devices. In more detail, the EMMs related to a specific type of IoT device are combined with EMMs of the same type of IoT device and therefore, a trusted EMM is produced for each different type of IoT device in the blockchain network. All the different trusted EMMs are stored in the blockchain and each IoT device utilizes the corresponding trusted EMM to perform anomaly detection. Thus, when an agent identifies a malicious activity, intelligence regarding this activity is shared with the rest of the agents in the blockchain network and similar types of activities trigger an alert from the whole system.

Finally, the proposed CIoTA framework is evaluated in an IoT simulation testbed which comprises 48 Raspberry Pis. The evaluation results have showed that the executed attacks could be adequately detected. Moreover, it is noted that a greater number of merged trained models could achieve a faster and stronger consensus.

Preuveneers et al. [58] proposed a permissioned blockchain-based federated deep learning method. The objectives were (1) to improve the detection of malicious behavior by employing federated learning techniques, and (2) to defend against potential poisoning attacks on the training datasets by employing blockchain. The authors evaluate their approach in a realistic use case involving multiple interconnected devices. They assume a network intrusion detection scenario with multiple interconnected devices. Regarding the machine learning model for performing intrusion detection, autoencoders were used as one-class classification neural networks for unsupervised anomaly detection. It is also assumed that training data were available only for representing the normal baseline behavior of the benign traffic.

**Figure 13:**
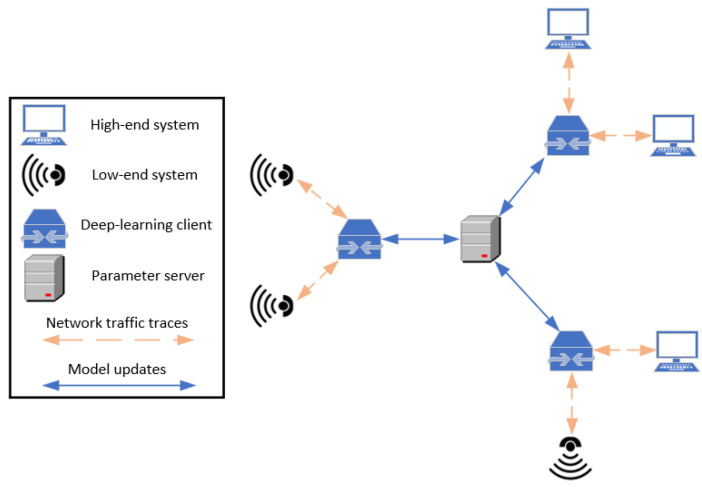
Federated learning in a network of devices.

Figure 13 demonstrates how federated learning is performed in a network of devices, as mentioned by the authors. Each client (i.e., deep learning client) acts as a gateway for a number of connected monitored systems (i.e., high-end systems, low-end systems), trains/updates a local model (i.e., autoencoder) based only on a dataset that originates from the network traffic traces of the connected devices. All the locally updated models of the clients have the same structure (i.e., number of layers and neurons per layer). Thus, the locally updated models can be combined by averaging their weights in order to produce a global updated model for the whole system. The aggregation of the weights to produce the global updated model is performed by the parameter server.

In this case, an adversary could try to compromise the parameter server and poison the global updated model by providing malicious model updates back to the clients. Thus, the authors replaced the parameter server with a blockchain. All the clients participate in this blockchain, and they record their locally updated models. Each client can then access the locally updated models from the blockchain and produce the global updated model without the need for a parameter server. The authors implement their proof-of-concept and their performance evaluation results show that the blockchain provided full transparency over the distributed training process, and the performance impact and network overhead of blockchain on the federated learning is limited.

**Figure 14:**
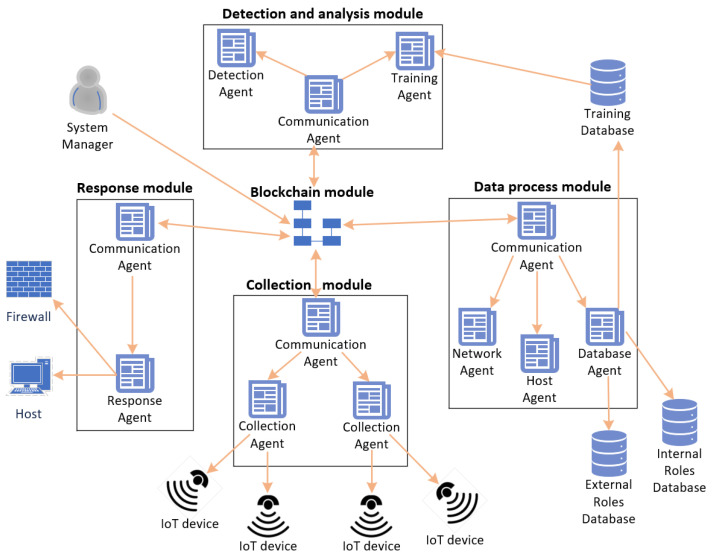
Architecture of the proposed system in [59].

Liang et al. at [59] propose an intrusion detection system for IoT devices based on blockchain and multi-agent systems. The proposed system is used to discover attacks from current network traffic and to monitor the details of the IoT network condition. The proposed system comprises 5 modules: (1) data collection module, (2) data process module, (3) detection and analysis module, (4) response module, and (5) blockchain smart contract module. Figure 14 demonstrates the architecture of the proposed system.

Firstly, the data collection module employs a collection agent and a communication agent and is responsible for gathering data from the monitored IoT devices. Secondly, the data process module employs a communication agent, a network agent or a host agent or a database agent. The data process module is responsible for performing data pre-processing on the collected data, including missing value processing, data integration, and data standardization. In addition, the data process module performs the first attack detection on the collected data, based on feature classification. Then, the collected data are sent to the detection and analysis module.

The detection and analysis module are responsible for detecting malicious patterns on unlabeled data and training a detection model based on labeled data. The detection and analysis module utilizes a communication agent, a detection agent, and a training agent. The results regarding the training of the model and the intrusion detection have been performed on unlabeled data are sent to the response module. The response module generates data visualization charts for the users according to the administrator’s response plan and performs firewall and host security changes according to the commands of the communication agent.

Finally, the blockchain module is responsible for implementing the blockchain which records all the actions of the communication agents residing in the data collection module, the data process module, the detection and analysis module, and the response module. The blockchain ledger containing all the communication records is replicated and shared among all the communication agents of the system. The communication agents run the consensus algorithm to validate a transaction before including it in the ledger. The authors have used the NSL-KDD dataset to test their system, and the experimental results have showed the efficiency of deep learning algorithms when detecting attacks from the transport layer. Furthermore, the source code of this system was released and can be download from Github [60].

Based on the works that were presented above, it is worthwhile observing that two main different approaches are described regarding how blockchain could be employed and leveraged in the context of intrusion detection for IoT networks. The first approach followed in [54,59] assumes the existence of an IoT network and of a CIDS that protects the IoT network and its IoT devices. An IDS runs on each IoT device of the IoT network and produces alert data. In addition, it is assumed that the various IDSs are a part of a CIDS that utilizes all the produced alert data in order to accurately detect intrusions in the IoT network. In this case, the use of blockchain is proposed in order to function as a distributed ledger that records the alert data produced by the IDSs running on the IoT devices of an IoT network. The action of recording the alert data of every participating IDS provides accountability and tamper-resistance to the CIDS that protects the IoT network. Therefore, the alert data are more trusted, and this, in turn, enhances the intrusion detection accuracy of the CIDS and the security provided to the IoT network. Moreover, it is important to consider the requirements of this first approach and thus, we present these requirements briefly in Table 6.

On the other hand, the second approach followed in [57,58] assumes the existence of an IoT network and of a machine learning-based IDS that protects the IoT network and its IoT devices. The IDS performs its operations in a federated learning environment where machine-learning (ML) models are trained in each separate IoT device, and the resulting trained ML models are aggregated by a central node. The aggregation of the trained ML models leads to the creation of a global trained ML model, which is then utilized by the IoT devices in order to perform intrusion detection. In this case, the integration of blockchain aims to support the training process by recording all the separately trained ML models in a distributed ledger shared among the IoT devices of the IoT network. Then, each IoT device can aggregate the trained ML models and produce the global trained ML model. Therefore, the aggregation process of the trained ML models becomes decentralized and potential SPoFs are removed. Additionally, it becomes more difficult for an intruder to poison the global trained ML model in order to evade detection, and thus, the produced global trained ML model is more trusted. Furthermore, it is worthwhile to consider the requirements of this second approach and thus, we present these requirements briefly in Table 6, along with the requirements of the first approach. The requirements of both approaches should be taken into consideration when they are adopted as security countermeasures into IoMT edge networks.

## 5. Conclusions

This paper focused on the investigation of the adoption of blockchain technology in the design and implementation of novel security mechanisms that will enhance the security of IoMT edge networks for healthcare monitoring, since blockchain has been foreseen by industry and the research community as a disruptive technology that can play a significant role in (a) securing IoMT devices, and (b) resisting unauthorized access during data transmission (i.e., tamper-proof transmission of medical data). Nevertheless, despite the fact that several blockchain-based security mechanisms have already been proposed in the literature for different types of IoT edge networks, there is a lack of blockchain-based security mechanisms for IoMT edge networks, and thus more effort is required to be put on the design and development of security mechanisms relying on blockchain technology for such networks. Therefore, in this paper, our focus was to examine the following two types of blockchain-based security mechanisms: (a) the very few existing ones specifically designed for IoMT edge networks, and (b) those designed for other types of IoT edge networks but could be possibly adopted in IoMT edge networks due to similar capabilities and technical characteristics between other types of IoT edge networks and IoMT edge networks. In particular, our target was to provide a foundation for organizing research efforts towards the design and development of reliable blockchain-based blockchain-based security mechanisms ensuring authentication and authorization as well as implementing AIDSs for IoT edge networks. As future work, we plan to take into consideration the outcome of this work, in terms of the strengths and weaknesses of the examined blockchain-based security mechanisms, and design novel reliable and efficient blockchain-based security mechanisms, ensuring authentication and authorization as well as implementing AIDSs for IoT edge networks. Then, we will conduct security analysis of the designed blockchain-based security mechanisms in order to evaluate them, in terms of the achieved security level, and select the most secure for implementation. Finally, the implemented blockchain-based security mechanisms will be evaluated based on their computational cost, communication overhead, and storage overhead.

## Figures and Tables

**Table 1:** Comparison of consensus protocols.

Consensus Mechanism	Type of Blockchain Used	Advantages	Limitations
PoW	public, permissionless	high security, malicious node tolerant	not efficient, high energy consumption, high computation cost
PoS	public, permissionless	power efficient, high security	concept of “stake” is not applicable in IoT
PBFT	permissioned	high throughput, low computational overhead	susceptible to Sybil attacks, poor scalability

**Table 2:** Comparison of various blockchain-based authentication mechanisms.

Reference	Type of Blockchain and Blockchain Platform	Advantages	Limitations	Implementation Parameters	Future Work
[38]	Permissioned, Hyperledger Fabric	decentralization, simplicity, general application	-	Raspberry Pi, Hyperledger Fabric	Management of IoT sensors data
[39]	Public, Ethereum Blockchain	decentralization, reduces latency, compliant to security requirements	transaction time delay and high energy consumption due to Ethereum properties	Ganache-cli, Ethereum emulator	Development of a lightweight consensus protocol for better results in terms of energy consumption
[36]	Ethereum Blockchain	decentralization, device credibility, patching	needs credible users	Geth client, Ethereum, Ubuntu VM	Investigate zero-knowledge proof encryption, real implementation
[40]	Public, Ethereum Blockchain	decentralization, scalability, resistant to attacks	not adapted to real time applications, no initialization phase, limited cryptocurrency rate	HP laptop- Ubuntu 14.04, Raspberry Pi–Rasbian 4.9.41, Ethereum	Controlled communication between bubbles, implementation of mechanism, design of a protocol for miner optimization
[41]	Private, Hyperledger fabric 1.4	suitable for password-based, certificate-based biotechnology- based authentication	high time complexity	virtual box 5.2.8, Ubuntu 16.4 server client Windows 10 pro intel i5-6200 2.30 Ghz CPU	-
[42]	private, JUICE blockchain platform	decentralization, anonymity	-	Ubuntu 16.04, Intel Core i7-6700 CPU 3.40 GHZ, 3-GB RAM, nginx-1.11.3, truffle-4.1.13, JUICE-client	Consider the attribute-based cryptographic approach in order to achieve better access control
[43]	-	authentication and confidentiality of data sharing		-	Anonymous authentication of IoT
[44]	Private Blockchain	decentralization, scalability	evaluation results are theoretical	Ethernet workshop	-
[37]	Private, Hyperledger Fabric	decentralization, data privacy, data integrity, SSI	-	-	Implementation of the designed mechanism in real life applications

**Table 3:** Blockchain-based Authorization Mechanisms in IoT networks.

Reference	Guarantees	Drawbacks
[53]	Decentralization	Limited in Protected Health Information Storage systems
	Availability	
	Confidentiality	
	Integrity	
	Immutability	
[45]	Lightweight, scalable, decentralized, and fine-grained access control solution for large-scale IoT systems.	Every domain involves a domain owner, which is a centralized entity; this might cause issues such as single point of failure, bottleneck, performance degradation, etc.
		A token is stored on the Blockchain which is visible in every participant; this will raise privacy issues.
[47]	More fine-grained access control and more flexible token management compared with existing capability-based AC schemes.	No results on the feasibility of the proposed scheme under a IoT healthcare system model, which involves several subjects such as users, doctors, nurses, etc.
	Experiments based on a local Ethereum blockchain demonstrated the feasibility of the scheme in large-scale IoT systems.	
	Promising to achieve dynamic and fine-grained access control as ABAC introduces context information and the attributes of subjects and objects into its access control policies. More accurate access control in sensitive applications such as Healthcare by including sufficient attributes. Reduces the burden of maintenance, as access policies can be changed by simply changing the attribute values without the need to change the underlying subject–object relationships.	Although the prototype demonstrates the feasibility of the proposed framework, it can hardly reflect the performance of the framework in large-scale IoT applications such as Healthcare applications. The authors consider as future work the implementation of the proposed framework in environments with larger scales.

**Table 4:** Requirements for the design of an effective and trustworthy CIDS mentioned in [54].

Requirement	Description
Accountability	Participating nodes must be held responsible for their actions.
Integrity	The integrity of the alert data should be guaranteed, since the accuracy of the detection depends on the alert data.
Resilience	The existence of single-points-of-failure (SPoFs) and the dependence of the system’s normal operations on a small group of nodes should be avoided.
Consensus	The proposed system needs to be able to reach a consensus regarding both the quality of individual alert data and the trustworthiness of each participating node.
Scalability	The proposed system needs to be scalable to a large number of participating nodes.
Minimum Overhead	The proposed system should incur minimum communication and computation overhead.
Privacy	The proposed system should provide the participating nodes with the ability to keep their alert data private and to selectively disclose alert data as they wish. Simultaneously, the requirements related to accountability and data integrity should still hold.

**Table 5:** Summary of implementation options for each design consideration mentioned in [54].

Design Consideration	Implementation Options
Governance of the distributed ledger	1. Public (permissionless) blockchain
	2. Consortium (permissioned) blockchain
Consensus algorithm	1. Proof-of-Work/Proof-of-Stake designs
	2. Practical Byzantine Fault Tolerance (PBFT) designs
Peers participating in the consensus algorithm	1. All nodes of CIDS participate.
	2. Only a subset of nodes of CIDS participate.
Detail of alert data during the dissemination process	1. Exchange of raw alert data
	2. Exchange of compact representations of the alert data (e.g., bloom filters [55,56])
	3. Hybrid (proposed by the authors): exchange of compact representations in the Consensus layer, and exchange of raw alert data in the Alert Exchange layer.
Data encryption in the Consensus layer	1. Symmetric key cryptography and distributing keys to specific participants.
	2. Exchange of compact representations of the alert data (e.g., bloom filters)

**Table 6:** Requirements of the two approaches described in [54,57,58,59] for blockchain-based IDS in IoT networks.

Approach	Requirements
Record the alert data produced by IDSs	(1) The IoT devices participating in the blockchain network should possess enough computational resources to run the consensus protocol of the blockchain network.
	(2) The IoT devices participating in the blockchain network should possess enough communication bandwidth to run the consensus protocol of the blockchain network.
	(3) The delay related to the inclusion of newly produced alert data to the blockchain ledger should be kept to a minimum. For this purpose, instead of raw alert data, hashes of the alert data can be stored in the distributed ledger.
Support and enhance the federated learning IDS	(1) The IoT devices participating in the blockchain network should possess enough computational resources to run the consensus protocol of the blockchain network. (2) The IoT devices participating in the blockchain network should possess enough computational resources to perform the aggregation of the separately trained ML models and produce the global trained ML model.
	(3) The IoT devices participating in the blockchain network should possess enough communication bandwidth to run the consensus protocol of the blockchain network.
	(4) The delay related to the creation of the global trained model should be kept to a minimum. For this purpose, the separately trained ML model could be stored in the distributed ledger in a compressed form.
